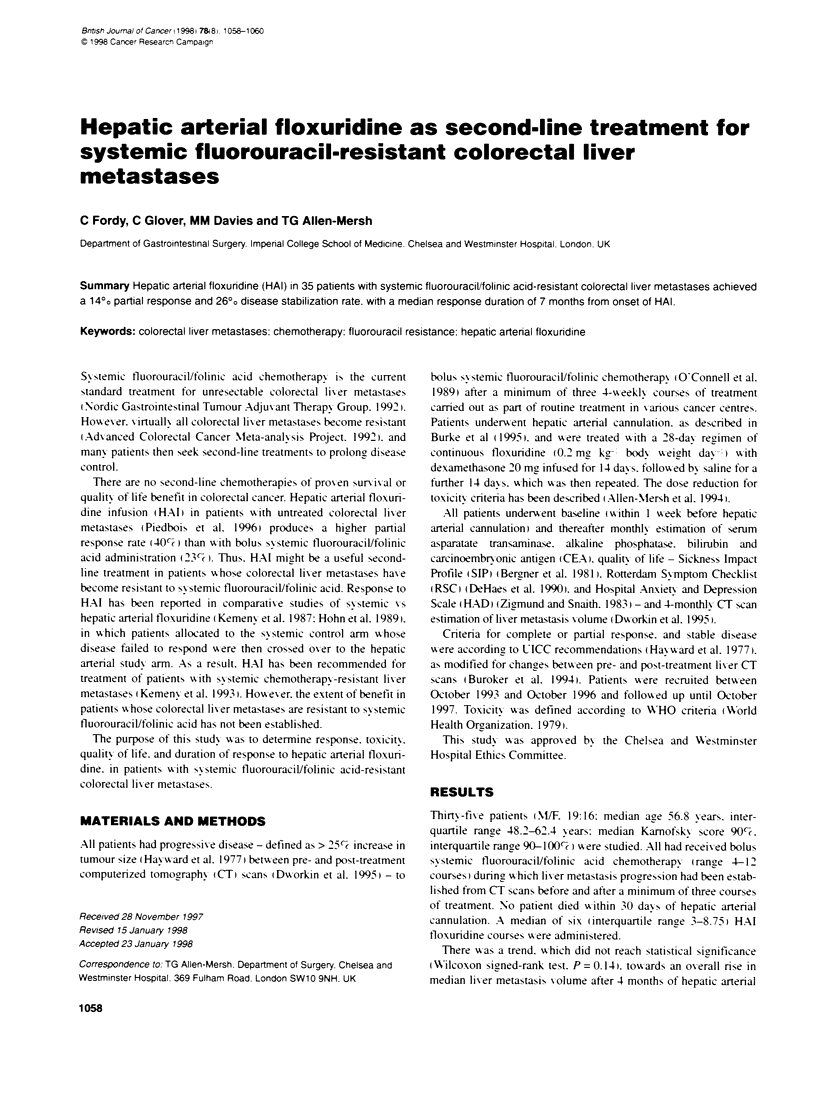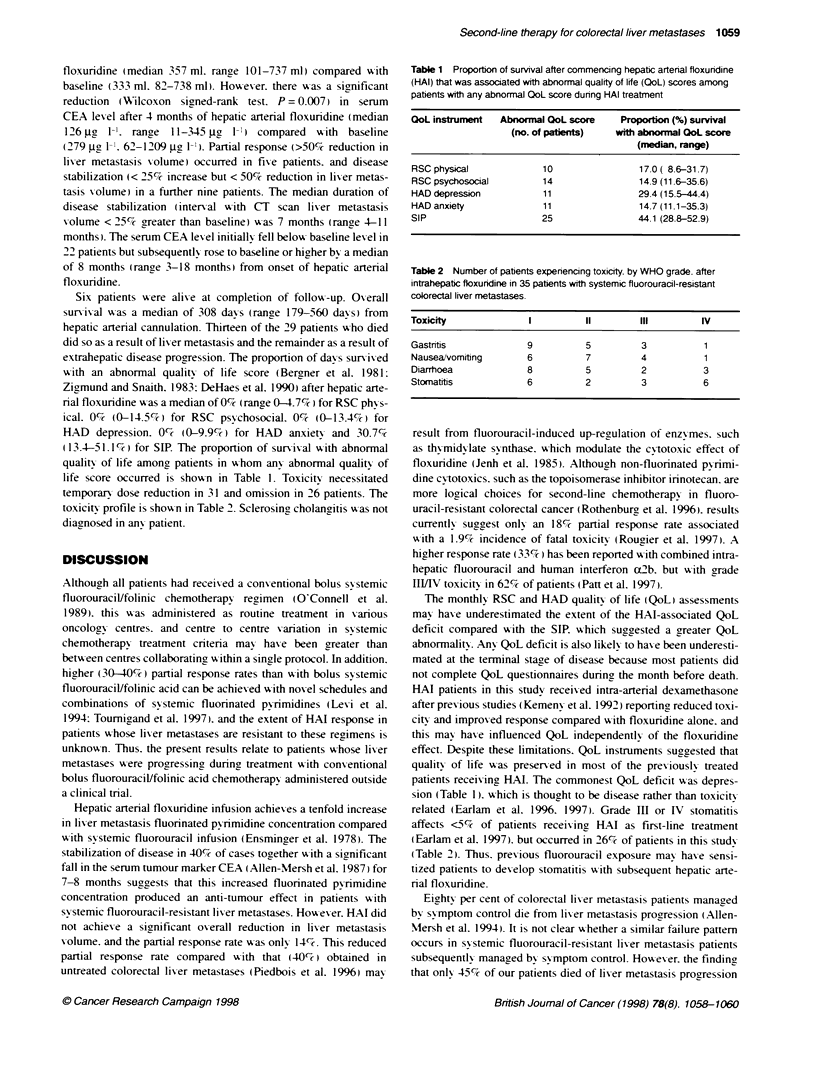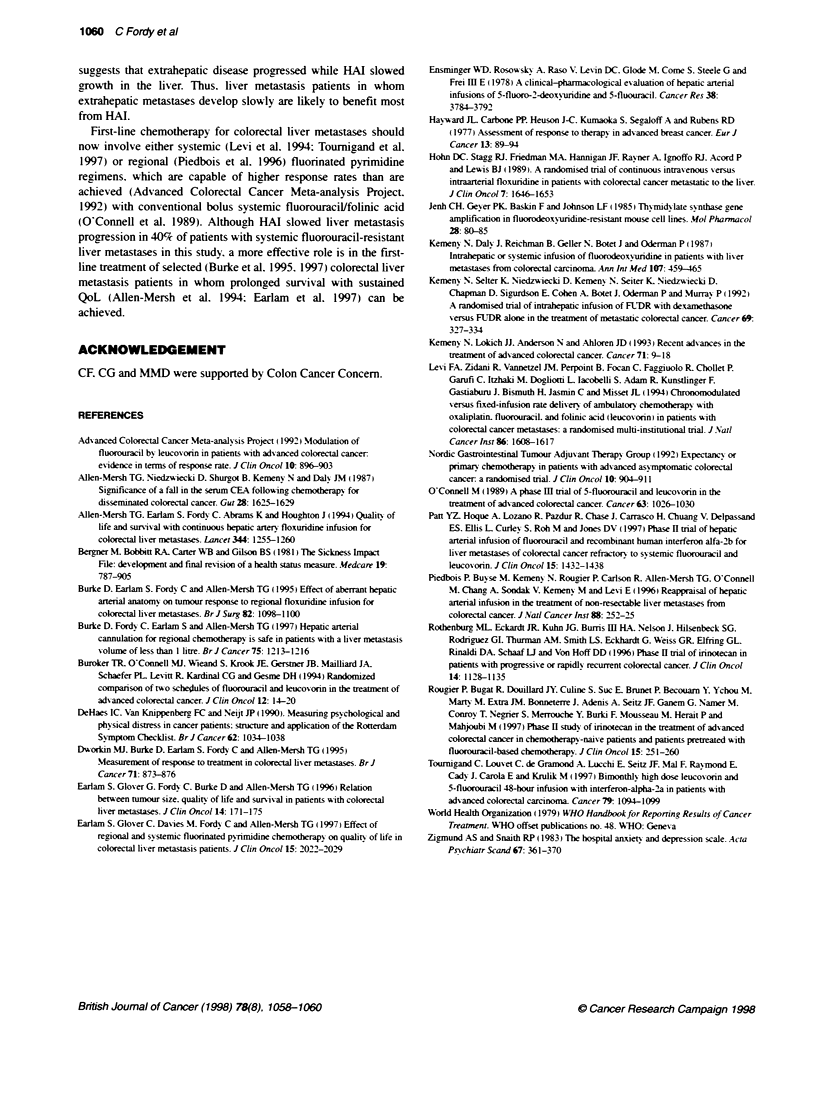# Hepatic arterial floxuridine as second-line treatment for systemic fluorouracil-resistant colorectal liver metastases.

**DOI:** 10.1038/bjc.1998.627

**Published:** 1998-10

**Authors:** C. Fordy, C. Glover, M. M. Davies, T. G. Allen-Mersh

**Affiliations:** Department of Gastrointestinal Surgery, Imperial College School of Medicine, Chelsea and Westminster Hospital, London, UK.

## Abstract

Hepatic arterial floxuridine (HAI) in 35 patients with systemic fluorouracil/folinic acid-resistant colorectal liver metastases achieved a 14% partial response and 26% disease stabilization rate, with a median response duration of 7 months from onset of HAI.


British Journal of Cancer (1998) 78(8), 1058–1060
© 1998 Cancer Research Campaign

### Hepatic arterial floxuridine as second-line treatment for systemic fluorouracil-resistant colorectal liver metastases

**C Fordy, C Glover, MM Davies and TG Allen-Mersh**

Department of Gastrointestinal Surgery. Imperial College School of Medicine. Chelsea and Westminster Hospital. London. UK

**Summary** Hepatic arterial floxuridine (HAI) in 35 patients with systemic fluorouracil/folinic acid-resistant colorectal liver metastases achieved a 14% partial response and 26% disease stabilization rate. with a median response duration of 7 months from onset of HAI.

**Keywords:** colorectal liver metastases: chemotherapy: fluorouracil resistance: hepatic arterial floxuridine

Systemic fluorouracil/folinic acid chemotherapy is the current standard treatment for unresectable colorectal liver metastases (Nordic Gastrointestinal Tumour Adjuvant Therapy Group. 1992). However. virtually all colorectal liver metastases become resistant (Advanced Colorectal Cancer Meta-analysis Project. 1992). and many patients then seek second-line treatments to prolong disease control.

There are no second-line chemotherapies of proven survival or quality of life benefit in colorectal cancer. Hepatic arterial floxuridine infusion (HAI) in patients with untreated colorectal liver metastases (Piedbois et al. 1996) produces a higher partial response rate (40%) than with bolus systemic fluorouracil/folinic acid administration (23%). Thus. HAI might be a useful second-line treatment in patients whose colorectal liver metastases have become resistant to systemic fluorouracil/folinic acid. Response to HAI has been reported in comparative studies of systemic vs hepatic arterial floxuridine (Kemeny et al. 1987: Hohn et al. 1989). in which patients allocated to the systemic control arm whose disease failed to respond were then crossed over to the hepatic arterial study arm. As a result. HAI has been recommended for treatment of patients with systemic chemotherapy-resistant liver metastases (Kemeny et al. 1993). However. the extent of benefit in patients whose colorectal liver metastases are resistant to systemic fluorouracil/folinic acid has not been established.

The purpose of this study was to determine response. toxicity. quality of life. and duration of response to hepatic arterial floxuridine. in patients with systemic fluorouracil/folinic acid-resistant colorectal liver metastases.

### MATERIALS AND METHODS

All patients had progressive disease – defined as > 25% increase in tumour size (Hayward et al. 1977) between pre- and post-treatment computerized tomography (CT) scans (Dworkin et al. 1995) – to bolus systemic fluorouracil/folinic chemotherapy (O'Connell et al. 1989) after a minimum of three 4-weekly courses of treatment carried out as part of routine treatment in various cancer centres. Patients underwent hepatic arterial cannulation. as described in Burke et al (1995). and were treated with a 28-day regimen of continuous floxuridine (0.2 mg $kg^{-1}$ body weight $day^{-1}$) with dexamethasone 20 mg infused for 14 days. followed by saline for a further 14 days. which was then repeated. The dose reduction for toxicity criteria has been described (Allen-Mersh et al. 1994).

All patients underwent baseline (within 1 week before hepatic arterial cannulation) and thereafter monthly estimation of serum asparatate transaminase. alkaline phosphatase. bilirubin and carcinoembryonic antigen (CEA). quality of life – Sickness Impact Profile (SIP) (Bergner et al. 1981). Rotterdam Symptom Checklist (RSC) (DeHaes et al. 1990). and Hospital Anxiety and Depression Scale (HAD) (Zigmund and Snaith. 1983) – and 4-monthly CT scan estimation of liver metastasis volume (Dworkin et al. 1995).

Criteria for complete or partial response. and stable disease were according to UICC recommendations (Hayward et al. 1977). as modified for changes between pre- and post-treatment liver CT scans (Buroker et al. 1994). Patients were recruited between October 1993 and October 1996 and followed up until October 1997. Toxicity was defined according to WHO criteria (World Health Organization. 1979).

This study was approved by the Chelsea and Westminster Hospital Ethics Committee.

### RESULTS

Thirty-five patients (M/F. 19:16: median age 56.8 years. interquartile range 48.2–62.4 years: median Karnofsky score 90%. interquartile range 90–100%) were studied. All had received bolus systemic fluorouracil/folinic acid chemotherapy (range 4–12 courses) during which liver metastasis progression had been established from CT scans before and after a minimum of three courses of treatment. No patient died within 30 days of hepatic arterial cannulation. A median of six (interquartile range 3–8.75) HAI floxuridine courses were administered.

There was a trend. which did not reach statistical significance (Wilcoxon signed-rank test. $P = 0.14$). towards an overall rise in median liver metastasis volume after 4 months of hepatic arterial

*Received 28 November 1997*
*Revised 15 January 1998*
*Accepted 23 January 1998*

*Correspondence to:* TG Allen-Mersh. Department of Surgery. Chelsea and Westminster Hospital. 369 Fulham Road. London SW10 9NH. UK

floxuridine (median 357 ml, range 101–737 ml) compared with baseline (333 ml, 82–738 ml). However, there was a significant reduction (Wilcoxon signed-rank test, $P = 0.007$) in serum CEA level after 4 months of hepatic arterial floxuridine (median 126 μg $l^{-1}$, range 11–345 μg $l^{-1}$) compared with baseline (279 μg $l^{-1}$, 62–1209 μg $l^{-1}$). Partial response (>50% reduction in liver metastasis volume) occurred in five patients, and disease stabilization (< 25% increase but < 50% reduction in liver metastasis volume) in a further nine patients. The median duration of disease stabilization (interval with CT scan liver metastasis volume < 25% greater than baseline) was 7 months (range 4–11 months). The serum CEA level initially fell below baseline level in 22 patients but subsequently rose to baseline or higher by a median of 8 months (range 3–18 months) from onset of hepatic arterial floxuridine.

Six patients were alive at completion of follow-up. Overall survival was a median of 308 days (range 179–560 days) from hepatic arterial cannulation. Thirteen of the 29 patients who died did so as a result of liver metastasis and the remainder as a result of extrahepatic disease progression. The proportion of days survived with an abnormal quality of life score (Bergner et al. 1981; Zigmund and Snaith, 1983; DeHaes et al. 1990) after hepatic arterial floxuridine was a median of 0% (range 0–4.7%) for RSC physical, 0% (0–14.5%) for RSC psychosocial, 0% (0–13.4%) for HAD depression, 0% (0–9.9%) for HAD anxiety and 30.7% (13.4–51.1%) for SIP. The proportion of survival with abnormal quality of life among patients in whom any abnormal quality of life score occurred is shown in Table 1. Toxicity necessitated temporary dose reduction in 31 and omission in 26 patients. The toxicity profile is shown in Table 2. Sclerosing cholangitis was not diagnosed in any patient.

**Table 1** Proportion of survival after commencing hepatic arterial floxuridine (HAI) that was associated with abnormal quality of life (QoL) scores among patients with any abnormal QoL score during HAI treatment

| QoL instrument | Abnormal QoL score (no. of patients) | Proportion (%) survival with abnormal QoL score (median, range) |
|---|---|---|
| RSC physical | 10 | 17.0 ( 8.6–31.7) |
| RSC psychosocial | 14 | 14.9 (11.6–35.6) |
| HAD depression | 11 | 29.4 (15.5–44.4) |
| HAD anxiety | 11 | 14.7 (11.1–35.3) |
| SIP | 25 | 44.1 (28.8–52.9) |

**Table 2** Number of patients experiencing toxicity, by WHO grade, after intrahepatic floxuridine in 35 patients with systemic fluorouracil-resistant colorectal liver metastases.

| Toxicity | I | II | III | IV |
|---|---|---|---|---|
| Gastritis | 9 | 5 | 3 | 1 |
| Nausea/vomiting | 6 | 7 | 4 | 1 |
| Diarrhoea | 8 | 5 | 2 | 3 |
| Stomatitis | 6 | 2 | 3 | 6 |

### DISCUSSION

Although all patients had received a conventional bolus systemic fluorouracil/folinic chemotherapy regimen (O'Connell et al. 1989), this was administered as routine treatment in various oncology centres, and centre to centre variation in systemic chemotherapy treatment criteria may have been greater than between centres collaborating within a single protocol. In addition, higher (30–40%) partial response rates than with bolus systemic fluorouracil/folinic acid can be achieved with novel schedules and combinations of systemic fluorinated pyrimidines (Levi et al. 1994; Tournigand et al. 1997), and the extent of HAI response in patients whose liver metastases are resistant to these regimens is unknown. Thus, the present results relate to patients whose liver metastases were progressing during treatment with conventional bolus fluorouracil/folinic acid chemotherapy administered outside a clinical trial.

Hepatic arterial floxuridine infusion achieves a tenfold increase in liver metastasis fluorinated pyrimidine concentration compared with systemic fluorouracil infusion (Ensminger et al. 1978). The stabilization of disease in 40% of cases together with a significant fall in the serum tumour marker CEA (Allen-Mersh et al. 1987) for 7–8 months suggests that this increased fluorinated pyrimidine concentration produced an anti-tumour effect in patients with systemic fluorouracil-resistant liver metastases. However, HAI did not achieve a significant overall reduction in liver metastasis volume, and the partial response rate was only 14%. This reduced partial response rate compared with that (40%) obtained in untreated colorectal liver metastases (Piedbois et al. 1996) may result from fluorouracil-induced up-regulation of enzymes, such as thymidylate synthase, which modulate the cytotoxic effect of floxuridine (Jenh et al. 1985). Although non-fluorinated pyrimidine cytotoxics, such as the topoisomerase inhibitor irinotecan, are more logical choices for second-line chemotherapy in fluorouracil-resistant colorectal cancer (Rothenburg et al. 1996), results currently suggest only an 18% partial response rate associated with a 1.9% incidence of fatal toxicity (Rougier et al. 1997). A higher response rate (33%) has been reported with combined intrahepatic fluorouracil and human interferon α2b, but with grade III/IV toxicity in 62% of patients (Patt et al. 1997).

The monthly RSC and HAD quality of life (QoL) assessments may have underestimated the extent of the HAI-associated QoL deficit compared with the SIP, which suggested a greater QoL abnormality. Any QoL deficit is also likely to have been underestimated at the terminal stage of disease because most patients did not complete QoL questionnaires during the month before death. HAI patients in this study received intra-arterial dexamethasone after previous studies (Kemeny et al. 1992) reporting reduced toxicity and improved response compared with floxuridine alone, and this may have influenced QoL independently of the floxuridine effect. Despite these limitations, QoL instruments suggested that quality of life was preserved in most of the previously treated patients receiving HAI. The commonest QoL deficit was depression (Table 1), which is thought to be disease rather than toxicity related (Earlam et al. 1996, 1997). Grade III or IV stomatitis affects <5% of patients receiving HAI as first-line treatment (Earlam et al. 1997), but occurred in 26% of patients in this study (Table 2). Thus, previous fluorouracil exposure may have sensitized patients to develop stomatitis with subsequent hepatic arterial floxuridine.

Eighty per cent of colorectal liver metastasis patients managed by symptom control die from liver metastasis progression (Allen-Mersh et al. 1994). It is not clear whether a similar failure pattern occurs in systemic fluorouracil-resistant liver metastasis patients subsequently managed by symptom control. However, the finding that only 45% of our patients died of liver metastasis progression

© Cancer Research Campaign 1998

suggests that extrahepatic disease progressed while HAI slowed growth in the liver. Thus, liver metastasis patients in whom extrahepatic metastases develop slowly are likely to benefit most from HAI.

First-line chemotherapy for colorectal liver metastases should now involve either systemic (Levi et al. 1994; Tournigand et al. 1997) or regional (Piedbois et al. 1996) fluorinated pyrimidine regimens, which are capable of higher response rates than are achieved (Advanced Colorectal Cancer Meta-analysis Project, 1992) with conventional bolus systemic fluorouracil/folinic acid (O'Connell et al. 1989). Although HAI slowed liver metastasis progression in 40% of patients with systemic fluorouracil-resistant liver metastases in this study, a more effective role is in the first-line treatment of selected (Burke et al. 1995, 1997) colorectal liver metastasis patients in whom prolonged survival with sustained QoL (Allen-Mersh et al. 1994; Earlam et al. 1997) can be achieved.

### ACKNOWLEDGEMENT

CF, CG and MMD were supported by Colon Cancer Concern.

### REFERENCES

Advanced Colorectal Cancer Meta-analysis Project (1992) Modulation of fluorouracil by leucovorin in patients with advanced colorectal cancer: evidence in terms of response rate. *J Clin Oncol* **10**: 896–903

Allen-Mersh TG, Niedzwiecki D, Shurgot B, Kemeny N and Daly JM (1987) Significance of a fall in the serum CEA following chemotherapy for disseminated colorectal cancer. *Gut* **28**: 1625–1629

Allen-Mersh TG, Earlam S, Fordy C, Abrams K and Houghton J (1994) Quality of life and survival with continuous hepatic artery floxuridine infusion for colorectal liver metastases. *Lancet* **344**: 1255–1260

Bergner M, Bobbitt RA, Carter WB and Gilson BS (1981) The Sickness Impact File: development and final revision of a health status measure. *Medcare* **19**: 787–905

Burke D, Earlam S, Fordy C and Allen-Mersh TG (1995) Effect of aberrant hepatic arterial anatomy on tumour response to regional floxuridine infusion for colorectal liver metastases. *Br J Surg* **82**: 1098–1100

Burke D, Fordy C, Earlam S and Allen-Mersh TG (1997) Hepatic arterial cannulation for regional chemotherapy is safe in patients with a liver metastasis volume of less than 1 litre. *Br J Cancer* **75**: 1213–1216

Buroker TR, O'Connell MJ, Wieand S, Krook JE, Gerstner JB, Mailliard JA, Schaefer PL, Levitt R, Kardinal CG and Gesme DH (1994) Randomized comparison of two schedules of fluorouracil and leucovorin in the treatment of advanced colorectal cancer. *J Clin Oncol* **12**: 14–20

DeHaes IC, Van Knippenberg FC and Neijt JP (1990). Measuring psychological and physical distress in cancer patients: structure and application of the Rotterdam Symptom Checklist. *Br J Cancer* **62**: 1034–1038

Dworkin MJ, Burke D, Earlam S, Fordy C and Allen-Mersh TG (1995) Measurement of response to treatment in colorectal liver metastases. *Br J Cancer* **71**: 873–876

Earlam S, Glover G, Fordy C, Burke D and Allen-Mersh TG (1996) Relation between tumour size, quality of life and survival in patients with colorectal liver metastases. *J Clin Oncol* **14**: 171–175

Earlam S, Glover C, Davies M, Fordy C and Allen-Mersh TG (1997) Effect of regional and systemic fluorinated pyrimidine chemotherapy on quality of life in colorectal liver metastasis patients. *J Clin Oncol* **15**: 2022–2029

Ensminger WD, Rosowsky A, Raso V, Levin DC, Glode M, Come S, Steele G and Frei III E (1978) A clinical–pharmacological evaluation of hepatic arterial infusions of 5-fluoro-2-deoxyuridine and 5-fluouracil. *Cancer Res* **38**: 3784–3792

Hayward JL, Carbone PP, Heuson J-C, Kumaoka S, Segaloff A and Rubens RD (1977) Assessment of response to therapy in advanced breast cancer. *Eur J Cancer* **13**: 89–94

Hohn DC, Stagg RJ, Friedman MA, Hannigan JF, Rayner A, Ignoffo RJ, Acord P and Lewis BJ (1989). A randomised trial of continuous intravenous versus intraarterial floxuridine in patients with colorectal cancer metastatic to the liver. *J Clin Oncol* **7**: 1646–1653

Jenh CH, Geyer PK, Baskin F and Johnson LF (1985) Thymidylate synthase gene amplification in fluorodeoxyuridine-resistant mouse cell lines. *Mol Pharmacol* **28**: 80–85

Kemeny N, Daly J, Reichman B, Geller N, Botet J and Oderman P (1987) Intrahepatic or systemic infusion of fluorodeoxyuridine in patients with liver metastases from colorectal carcinoma. *Ann Int Med* **107**: 459–465

Kemeny N, Selter K, Niedzwiecki D, Kemeny N, Seiter K, Niedzwiecki D, Chapman D, Sigurdson E, Cohen A, Botet J, Oderman P and Murray P (1992) A randomised trial of intrahepatic infusion of FUDR with dexamethasone versus FUDR alone in the treatment of metastatic colorectal cancer. *Cancer* **69**: 327–334

Kemeny N, Lokich JJ, Anderson N and Ahloren JD (1993) Recent advances in the treatment of advanced colorectal cancer. *Cancer* **71**: 9–18

Levi FA, Zidani R, Vannetzel JM, Perpoint B, Focan C, Faggiuolo R, Chollet P, Garufi C, Itzhaki M, Dogliotti L, Iacobelli S, Adam R, Kunstlinger F, Gastiaburu J, Bismuth H, Jasmin C and Misset JL (1994) Chronomodulated versus fixed-infusion rate delivery of ambulatory chemotherapy with oxaliplatin, fluorouracil, and folinic acid (leucovorin) in patients with colorectal cancer metastases: a randomised multi-institutional trial. *J Natl Cancer Inst* **86**: 1608–1617

Nordic Gastrointestinal Tumour Adjuvant Therapy Group (1992) Expectancy or primary chemotherapy in patients with advanced asymptomatic colorectal cancer: a randomised trial. *J Clin Oncol* **10**: 904–911

O'Connell M (1989) A phase III trial of 5-fluorouracil and leucovorin in the treatment of advanced colorectal cancer. *Cancer* **63**: 1026–1030

Patt YZ, Hoque A, Lozano R, Pazdur R, Chase J, Carrasco H, Chuang V, Delpassand ES, Ellis L, Curley S, Roh M and Jones DV (1997) Phase II trial of hepatic arterial infusion of fluorouracil and recombinant human interferon alfa-2b for liver metastases of colorectal cancer refractory to systemic fluorouracil and leucovorin. *J Clin Oncol* **15**: 1432–1438

Piedbois P, Buyse M, Kemeny N, Rougier P, Carlson R, Allen-Mersh TG, O'Connell M, Chang A, Sondak V, Kemeny M and Levi E (1996) Reappraisal of hepatic arterial infusion in the treatment of non-resectable liver metastases from colorectal cancer. *J Natl Cancer Inst* **88**: 252–25

Rothenburg ML, Eckardt JR, Kuhn JG, Burris III HA, Nelson J, Hilsenbeck SG, Rodriguez GI, Thurman AM, Smith LS, Eckhardt G, Weiss GR, Elfring GL, Rinaldi DA, Schaaf LJ and Von Hoff DD (1996) Phase II trial of irinotecan in patients with progressive or rapidly recurrent colorectal cancer. *J Clin Oncol* **14**: 1128–1135

Rougier P, Bugat R, Douillard JY, Culine S, Suc E, Brunet P, Becouarn Y, Ychou M, Marty M, Extra JM, Bonneterre J, Adenis A, Seitz JF, Ganem G, Namer M, Conroy T, Negrier S, Merrouche Y, Burki F, Mousseau M, Herait P and Mahjoubi M (1997) Phase II study of irinotecan in the treatment of advanced colorectal cancer in chemotherapy-naive patients and patients pretreated with fluorouracil-based chemotherapy. *J Clin Oncol* **15**: 251–260

Tournigand C, Louvet C, de Gramond A, Lucchi E, Seitz JF, Mal F, Raymond E, Cady J, Carola E and Krulik M (1997) Bimonthly high dose leucovorin and 5-fluorouracil 48-hour infusion with interferon-alpha-2a in patients with advanced colorectal carcinoma. *Cancer* **79**: 1094–1099

World Health Organization (1979) *WHO Handbook for Reporting Results of Cancer Treatment*. WHO offset publications no. 48. WHO: Geneva

Zigmund AS and Snaith RP (1983) The hospital anxiety and depression scale. *Acta Psychiatr Scand* **67**: 361–370

*© Cancer Research Campaign 1998*